# Burden of exposure to infectious bursal disease virus, infectious bronchitis virus, Newcastle disease virus, *Mycoplasma gallisepticum*, and intestinal parasites in introduced broiler chickens on the Galapagos

**DOI:** 10.1371/journal.pone.0203658

**Published:** 2018-09-24

**Authors:** Ashley B. R. Whitehead, Gary D. Butcher, Heather S. Walden, Viviana Duque, Marilyn Cruz, Jorge A. Hernandez

**Affiliations:** 1 College of Veterinary Medicine, University of Florida, Gainesville, Florida, United States of America; 2 Agencia de Regulación y Control de la Bioseguridad y Cuarentena para Galápagos, Santa Cruz, Galapagos, Ecuador; 3 Center for Latin American Studies, University of Florida, Gainesville, Florida, United States of America; 4 College of Public Health and Health Professions, University of Florida, Gainesville, Florida, United States of America; Tokat Gaziosmanpasa University, TURKEY

## Abstract

Diseases in introduced broilers can possibly spill over to wild birds on the Galapagos. Knowledge about the current burden of exposure to pathogens in broilers on the Galapagos is very limited. The objective of the study reported here was to measure the burden of exposure to infectious bursal disease virus (IBDV), infectious bronchitis virus (IBV), Newcastle disease virus (NDV), *Mycoplasma gallisepticum* (MG), and intestinal parasites in a sample of broiler chickens on 13 farms on Santa Cruz Island and San Cristobal Island in July 2017. Blood serum samples were tested for detection of antibodies to IBDV, IBV, NDV, and MG by using an IDEXX Enzyme-linked Immunosorbent Assay. In addition, fecal samples and pen bedding environmental samples were processed and analyzed for diagnosis of intestinal parasite eggs under a compound light microscope. The frequency of seropositive broilers to IBDV was 74/130 or 56% (95% CI = 48, 65%), to IBV was 27/130 or 20% (14, 28%), and to NDV was 1/130 or 0.7% (0.1, 4%). All broilers tested negative to MG antibodies. *Eimeria* spp. infection was common in study broilers. Finally, we observed interaction between broiler chickens and wild birds (finches) inside broiler pens, as well as the presence of backyard chickens inside property limits of study farms. This study produced evidence that exposure to IBDV, IBV, and intestinal parasites in broilers on Santa Cruz Island and San Cristobal Island is important. Study results are relevant because (i) they provide new baseline data on the burden of exposure to avian pathogens in broiler farms, (ii) justify the need to verify standard operating procedures in hatcheries that supply (non-vaccinated) day-old chicks to the Galapagos and (iii) to implement enhanced biosecurity standards on broiler chicken farms to mitigate risk of disease transmission between broilers, backyard poultry, and wild birds on the Galapagos.

## Introduction

The rapid expansion of the human population and tourism industry has created a demand for poultry products on the Galapagos in the past two decades. For example, the importation of one-day-old broiler chicks into Santa Cruz Island increased by 115% from 143,000 chicks in 2005 to 308,500 chicks in 2016. Currently, the number of broilers per farm varies from 500 to 9,000. The average duration of the production cycle is six weeks, when broilers reach a body weight of about 2.7 kg (6 lb). Broilers are harvested on the farm by attending personnel and sold chilled or frozen in the local market (e.g., meat shops, grocery stores, restaurants, tourist boats).

Introduced broilers are susceptible to avian pathogens that can possibly spill over to wild birds on the Galapagos. For example, infections with infectious bursal disease (birnavirus) in broiler chickens can potentially spillover to lava gulls and Galapagos penguins [1]. Infections with infectious bronchitis virus (coronavirus) can be transmitted to Galapagos doves [1]. Infections with Newcastle disease virus (paramyxovirus-1) can cause morbidity and mortality in the flightless cormorant, brown pelican, Galapagos penguin, lava gull, Galapagos finches, mockingbirds, and Galapagos pintail [1,2]. In addition, *Mycoplasma gallisepticum* infections can cause morbidity and population declines in Darwin’s finches, mockingbirds, Galapagos doves, dark-billed cuckoos, and yellow warblers [1,2]. The use of vaccines in day-old chicks shipped to the Galapagos and after arrival is prohibited. This policy requires high biosecurity standards to mitigate risk of disease transmission between broilers, backyard chickens, and wild birds on the archipelago.

Knowledge of disease burden in introduced broilers on the Galapagos is limited to two studies. During 2001–2003, a study conducted on San Cristobal Island produced evidence of prior exposure to several pathogens in 72 broilers including: (i) infectious bursal disease virus, (42%); (ii) infectious bronchitis virus (46%); (iii) Newcastle disease virus (22%); and (iv) *M*. *gallisepticum* (7%) [1]. In 2005, a study on Santa Cruz Island revealed that the burden of previous exposure to these four pathogens in 88 broilers varied from 3% to 73% [2]. In both studies, indirect contact with infected backyard chickens or vaccination (although illegal) were suspected as potential risk factors associated with broilers demonstrating positive antibody titers to investigated pathogens. Finally, the study on San Cristobal did not test broilers for intestinal parasites [1], and the study on Santa Cruz did not find evidence of exposure to intestinal parasites in study broilers [2]. To our knowledge, no other studies have investigated or confirmed the burden of exposure to pathogens in broilers on San Cristobal and Santa Cruz since 2003 and 2005, respectively.

In October 2012, Ecuador’s Ministry of the Environment established the Agencia de Regulación y Control de la Bioseguridad y Cuarentena para Galápagos (Galapagos Biosecurity Agency) (ABG). An important mandate of the ABG is to regulate, control and prevent the introduction and dissemination of introduced species that represent a hazard to Galapagos native species and their habitat. An issue of concern is diseases in poultry that represent a health hazard to poultry, people, and wild birds on the archipelago. The objective of the study reported here was to produce new baseline data on the burden of exposure to infectious bursal disease virus (IBDV), infectious bronchitis virus (IBV), Newcastle disease virus (NDV), *M*. *gallisepticum* (MG) and intestinal parasites in a sample of introduced broilers on 13 farms on Santa Cruz Island and San Cristobal Island, Galapagos in July 2017. This information is important to support current ABG science-based policymaking efforts aimed at reducing the burden of diseases in broilers that can spill over to wild birds on the Galapagos.

## Materials and methods

This study received approval from the University of Florida’s Institute of Animal Care and Use Committee. The study was conducted during 10–14 July 2017 as part of a poultry farmers’ training workshop on biosecurity, diagnosis and risk management of diseases in broilers on Santa Cruz Island and San Cristobal Island, Galapagos. The workshop was organized by the ABG.

### Study population

In July 2017, there were 25 broiler farms on Santa Cruz (farm bird maximum capacity: 500 to 9000) and seven broiler farms on San Cristobal (2500 to 8000). On all farms, the source of broilers was one-day-old chicks supplied by four commercial chicken egg hatcheries in Guayaquil, Ecuador. Based on a current law and regulations for control of introduced species on the Galapagos (Libro VII del Regimen Especial: Galapagos, Capítulo III, Artículo 6 http://bioseguridadgalapagos.gob.ec/lista-de-productos-2/) the use of vaccines (e.g., against IBDV, IBV, NDV) in one-day-old chicks shipped to Galapagos, and after arrival, is prohibited. Local authorities do not approve the use of poultry vaccines because poultry are not native island species, and because the use of vaccines in introduced species requires vaccination guidelines supported by a risk assessment that must be approved by local authorities. Chicks are transported by commercial air carriers from Guayaquil to Baltra Island (then crossed by a small ferry to Santa Cruz Island and after by land transportation to the farm) or to San Cristobal.

Poultry pens are made of concrete or dirt floors, chicken-wire or wire mesh for fencing, galvanized metal roof, and tarp, roll-up side curtains for ventilation. Poultry pen bedding material is made out of wood shavings and is changed after each production cycle. Currently, there is no policy that prohibits the use of poultry litter as fertilizer on agricultural fields.

The average production cycle is six weeks, when broiler chicken body weight reaches about 2.7 kg. Broilers are fed using commercial diets and tap water. Farms use two to four feed formulations for different age groups (starter 1 and 2, grower, finisher diets). The first three formulations can include the use of anti-coccidials. Broilers are harvested on the farm by attending personnel. Harvested broilers are distributed chilled or frozen in the local market (restaurants, meat stores, tourist boats).

### Study farms and broilers

A non-random, convenience sample of 13 broiler farms was selected based on available funds, interest and willingness of farmers to participate. Six of 25 broiler chicken farms on Santa Cruz Island (1300 to 5200 broilers per farm) and all seven broiler chicken farms on San Cristobal Island (1050 to 6400 broilers per farm) were included. On each study farm, 10 clinically healthy broilers from one pen at harvesting age (six weeks) were selected by the farm manager and included. The sample size justification of 10 broilers per farm was based on input from ABG’s management office and farm managers, as well as on funding available for the study. On each study farm, all 10 broilers were offered for blood sample collection. In addition, two of the 10 broilers were harvested for a postmortem examination and collection of intestinal (fecal) samples as part of the training workshop.

### Biosecurity

All study farms practiced biosecurity control measures to prevent exposure to contaminated vehicles, equipment, clothing and footwear by visitors. Personal protective equipment including disposable plastic footwear, coveralls, and gloves were used by study personnel on each study farm. In addition, several study farm managers required the use of disposable facemasks and hairnets and that disposable footwear and the study truck be sprayed with a disinfectant prior to entering the premises.

Study farms had two or more poultry pens with broilers of different age groups. In addition, poultry pens were not 100% rodent- or native bird-proof. Finally, backyard poultry were present within farm property limits.

### Collection of blood and fecal samples

Approximately 2–3 ml of blood were collected from the ulnar vein using new, disposable 3 ml syringes (20-g 1-inch needle) on each study bird. Blood samples were poured into new, individual 10-ml vacutainer tubes (rubber stopper was removed from the tube before use to release the vacuum and reduce risk of red blood cell hemolysis). In addition, approximately 5 g of feces were collected from the intestinal tract of study birds. Finally, one environmental sample (broiler pen bedding) was collected from the same broiler pen used by study broilers. Fecal samples and environmental samples were placed in individual plastic whirl-bags. All samples were identified using a unique farm letter code, broiler number, or environmental sample number. Samples were stored in a cooler with ice packs at study farms and during transportation to a designated laboratory at ABG for processing the same day.

### Detection of antibodies against infectious bursal disease virus, infectious bronchitis virus, Newcastle disease virus, and *M*. *gallisepticum*

Blood serum samples were tested for detection of antibodies to IBDV, IBV, NDV, and MG by using an IDEXX Enzyme-linked Immunosorbent Assay (ELISA). Laboratory tests were conducted at the University of Georgia’s Poultry Diagnostic and Research Center in Athens, Georgia. Samples were classified as seropositive or seronegative to each pathogen of interest using antibody concentration cut-off points (i.e., IBVD S/P ratio = 0.50; IBV S/P ratio = 0.20; NDV S/P ratio = 0.20; MG S/P ratio = 0.20) recommended by the manufacturer (IDEXX IBD Ab Test Validation Data Report; IDEXX IBV Ab Test Validation Data Report IDEXX NDV Ab Test Validation Data Report; IDEXX MG Ab Test Validation Data Report). Using the HI test as gold standard, the relative sensitivity of the four tests = 100% in 10, 8, 11, and 30 experimentally vaccinated chickens, respectively. Similarly, the relative specificity of the four tests = 100% in 45, 45, 31, and 30 specific pathogen free chickens, respectively.

### Diagnosis of intestinal parasites

Fecal samples and environmental samples were processed and analyzed in a designated laboratory at ABG on Santa Cruz or San Cristobal. A fecal flotation was performed with approximately 1 g of feces from each sample using Sheather’s sugar solution (Sp. 1.25) [3]. Samples were allowed to set at room temperature for two hours with a cover glass. Next, samples were analyzed for diagnosis of intestinal parasite eggs under a compound light microscope with a total magnification of 400x.

### Data collection

On each study farm, the following data were collected: location (Santa Cruz or San Cristobal), number of broiler pens, number of broilers, breed (Ross, Cobb), number of employees, duration of production cycle (days), average mortality per cycle (%) in the last 12 months, and main cause of mortality in broilers reported by the farm manager.

### Data analysis

Descriptive statistics (median; minimum, maximum) were calculated for continuous variables, such: number of broilers on study farms, duration (days) of the production cycle, mortality (%) per cycle, and S/P ratios for each investigated pathogen by farm. The proportions of broilers classified as positive to antibody titers to IBDV, IBD, NDV, or MG were calculated by dividing the number of seropositive broilers to each pathogen of interest by the total number of tested broilers. Ninety-five percent confident intervals (95% CI) were calculated for each proportion using a statistical software program [4].

## Results

The number of pens on each farm varied from two to five (Table 1). Pens on each farm had broilers of different age groups. Median numbers of broilers on study farms in Santa Cruz and San Cristobal were 1950 (minimum = 1300, maximum = 5200) and 3000 (1050, 6400), respectively. Median duration of the production cycle on study farms was 42 days on Santa Cruz (42, 42) and 42 days on San Cristobal (42, 60). Median mortality (%) per cycle on study farms was 1.5% on Santa Cruz (0.9, 2.4) and 3.0% on San Cristobal (0.9, 5.0). The main causes of mortality in broilers reported by farm managers were dehydration during the first week of age or heat stress after the first three weeks of age (2/6 farms on Santa Cruz and 2/7 farms on San Cristobal) and heart attack during the fifth and sixth weeks of age (2/6 farms on Santa Cruz and 3/7 farms on San Cristobal).

**Table 1:** Study broiler farms on Santa Cruz Island and San Cristobal Island, Galapagos: July 2017.

Island	Farm	Pens	Employees	Maximum capacity (broilers)	Current population (broilers)	Breed	Production cycle (days)	Mortality per cycle (%)
Santa Cruz	A	4	3	6000	5200	Ross	42	1.6
	B	3	2	2100	1400	Cobb	42	1.4
	C	4	1	6000	2500	Cobb	42	2.4
	D	3	3	4200	4200	Ross	42	0.9
	E	2	1	2400	1400	Ross	42	1.0
	F	2	3	1900	1300	Cobb	42	2.0
San Cristobal	G	3	2	6000	4500	Cobb	60	3.0
	H	5	2	8000	6400	Cobb	42	0.9
	I	3	2	2500	1050	Cobb	42	1.8
	J	4	1	4000	3000	Cobb	42	3.0
	K	3	1	3000	2000	Cobb	42	5.0
	L	4	1	4400	3300	Cobb	42	3.6
	M	3	1	8000	1500	Cobb	45	3.0

All study farms had ≥ 1 broilers classified as seropositive to IBDV (Table 2). Overall, the frequency of broilers with positive antibody titers to IBDV was 26/60 or 43% (95% CI = 32, 56%) on Santa Cruz and 48/70 = 69% (57, 78%) on San Cristobal. In addition, three of six farms on Santa Cruz and one of seven farms on San Cristobal had ≥ 1 broilers classified as seropositive to IBV; the frequency of broilers with positive antibody titers to IBV was 22/60 or 37% (26, 49%) on Santa Cruz and 5/70 = 7% (3, 16%) on San Cristobal. One of six farms on Santa Cruz had one broiler classified as seropositive to NDV (S/P ratio = 0.25). All broilers tested negative to MG antibodies.

**Table 2:** Frequency of study broilers that tested positive to antibodies against infectious bursal disease virus (IBDV), infectious bronchitis virus (IBV), Newcastle disease virus (NDV), or M. gallisepticum (MG) on 13 study broiler farms.

Island	Farm	Broilers tested	Serology	IBDV	IBV	NDV	MG
Santa Cruz	A	10	Positive^1^	1	6	0	0
			S/P ratio^2^	0.03 (0.00, 0.41)	0.08 (0.05, 0.45)	0.06, (0.06, 0.07)	0.00 (0.00, 0.00)
	B	10	Positive	3	0	0	0
			S/P ratio	0.09 (0.01, 0.71)	0.01 (0.00, 0.07)	0.03 (0.00, 0.09)	0.01 (0.00, 0.07)
	C	10	Positive	2	0	0	0
			S/P ratio	0.07 (0.04, 0.39)	0.007 (0.00, 0.11)	0.004 (0.01, 0.11)	0.009 (0.00, 0.27)
	D	10	Positive	8	7	1	0
			S/P ratio	0.27 (0.05, 0.89)	0.25 (0.05, 2.17)	0.03 (0.00, 0.26)	0.01 (0.00, 0.26)
	E	10	Positive	10	9	0	0
			S/P ratio	0.38 (0.20, 0.83)	0.57 (0.14, 4.07)	0.07 (0.03, 0.13)	0.05 (0.02, 0.22)
			Positive	2	0	0	0
	F	10	S/P ratio	0.10 (0.01, 0.40)	0.00 (0.00, 0.00)	0.01 (0.00, 0.20)	0.00 (0.00, 0.05)
	Total	60	Positive	26 (43.3%)	22 (36.6%)	1 (1.6%)	0
San Cristobal	G	10	Positive	2	0	0	0
			S/P ratio	0.13 (0.06, 0.24)	0.00 (0.00, 0.03)	0.02 (0.00, 0.07)	0.02 (0.00, 0.05)
	H	10	Positive	10	0	0	0
			S/P ratio	1.46 (0.83, 2.63)	0.00 (0.00, 0.00)	0.02 (0.00, 0.07)	0.02 (0.00 0.05)
	I	10	Positive	7	5	0	0
			S/P ratio	0.33 (0.04, 0.54)	0.19 (0.02, 0.47)	0.04 (0.00, 0.09)	0.02 (0.00, 0.08)
	J	10	Positive	5	0	0	0
			S/P ratio	0.23 (0.06, 0.65)	0.00 (0.00, 0.02)	0.00 (0.00, 0.14)	0.02 (0.00, 0.10)
	K	10	Positive	9	0	0	0
			S/P ratio	0.52 (0.19, 1.20)	0.01 (0.00, 0.09)	0.02 (0.00, 0.13)	0.01 (0.00, 0.08)
	L	10	Positive	5	0	0	0
			S/P ratio	0.14 (0.01, 0.37)	0.00 (0.00, 0.06)	0.00 (0.00, 0.13)	0.00 (0.00, 0.04)
	M	10	Positive	10	0	0	0
			S/P ratio	0.39 (0.22, 0.65)	0.05 (0.03, 0.59)	0.01 (0.00, 0.45)	0.02 (0.00, 0.07)
	Total	70	Positive	48 (68.5%)	5 (7.1%)	0	0
	ALL	130	Positive	74 (56.9%)	27 (20.7%)	1 (0.7%)	0

^1^Positive: number of broilers that tested positive to antibodies against IBDV, IBV, NDV, or MG.

^2^S/P ratio (sample/positive ratio): data are reported as median (minimum, maximum)

**Fig 1:**
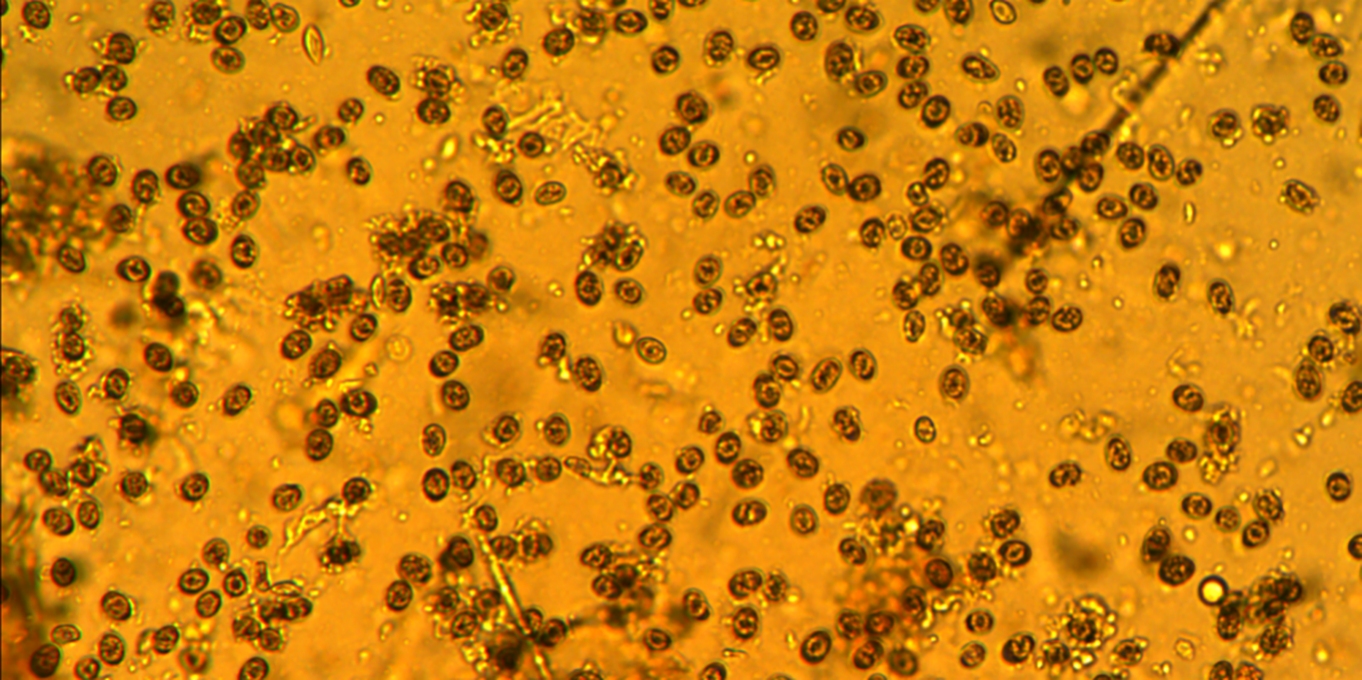
*Eimeria* spp. oocysts observed in intestinal (fecal) samples in one broiler chicken on Farm K in San Cristobal.

Overall, two of six farms on Santa Cruz and six of seven farms on San Cristobal had evidence of exposure to *Eimeria* spp (Table 3; Fig 1). In addition, two of 26 harvested broilers had intestinal tract lesions. On Santa Cruz (Farm A), one of two broilers examined contained mild lesions of the proximal duodenal mucosa (i.e., petechiae, white spots); however, no oocysts were observed on fecal flotation. On Farm F, one of two broilers examined had presence of blood and mucus in the cecum.

**Table 3:** Study broilers and farms with a positive diagnosis of *Eimeria* spp. in fecal samples and environmental samples collected from 13 broiler farms on Santa Cruz Island and San Cristobal Island, Galapagos: July 2017.

Island	Farm	Broiler 1*	Broiler 2*	Bed*
Santa Cruz	A	NPO**	NPO	NPO
	B	NPO	NPO	NPO
	C	No sample	NPO	NPO
	D	NPO	NPO	NPO
	E	*Eimeria* spp	*Eimeria* spp	*Eimeria* spp
	F	*Eimeria* spp	*Eimeria* spp	*Eimeria* spp
San Cristobal	G	NPO	NPO	*Eimeria* spp
	H	*Eimeria* spp	*Eimeria* spp	*Eimeria* spp
	I	*Eimeria* spp	*Eimeria* spp	*Eimeria* spp
	J	NPO	NPO	NPO
	K	*Eimeria* spp	*Eimeria* spp	Eimeria spp
	L	NPO	*Eimeria* spp	*Eimeria* spp
	M	*Eimeria* spp	*Eimeria* spp	*Eimeria* spp

*On each study farm, fecal samples from two harvested broilers and one environmental (pen bedding) sample were collected and examined for diagnosis of intestinal parasites.

**NPO = No parasites observed.

## Discussion

This study produced evidence that exposure to IBDV, IBV, and intestinal parasites in broilers on Santa Cruz Island and San Cristobal Island is important. It is not clear whether the observed exposure to IBDV or IBD indicates natural exposure or vaccination through accidental or illegal means. *Eimeria* spp. infection was common in broilers although this finding does not represent a hazard to native wild birds. In addition, we observed interaction between broilers and wild birds (finches) inside broiler pens, as well as the presence of backyard chickens inside property limits of study farms. Study results are relevant because (i) they provide new baseline data on the burden of exposure to avian pathogens in broiler farms, (ii) justify the need to verify standard operating procedures in hatcheries that supply (non-vaccinated) day-old chicks to the Galapagos and (iii) to implement enhanced biosecurity standards on broiler chicken farms to mitigate risk of disease transmission between broilers, backyard poultry, and wild birds on the Galapagos.

All study farms on Santa Cruz and San Cristobal had ≥ 1 broilers with positive antibody titers against IBDV. In addition, three of six farms on Santa Cruz and one of seven farms on San Cristobal had ≥ 1 broilers with positive antibody titers against IBV. Possible explanations for the observed exposure to these two pathogens can be: (i) accidental vaccination (e.g., cross-contamination by spray vaccination in hatchery); or (ii) indirect transmission between infected backyard chickens and susceptible broilers (via farm personnel or use of equipment contaminated with field IBDV and/or IBV strains circulating in backyard chickens nearby study farms); although a limitation in this study was that backyard chickens were not investigated.

Two previous studies have produced serologic evidence of exposure to IBDV and IBV in backyard chickens and broilers on the Galapagos. One study on San Cristobal reported that exposure to IBDV was high in both backyard chickens (9/25 = 36%; 95% CI = 20, 55%) during 2001–2003 and broilers (30/72 = 42%; 31, 53%) in November 2003 [1]. Furthermore, that study reported that exposure to IBV was high in both backyard chickens (6/8 = 75%; 41, 93%) and broilers (33/72 = 46%; 35, 57%) during the same time periods; however, the numbers of farms with one or more broilers classified as seropositive to IBDV or IBV were not reported. Another study produced evidence that burden of exposure to IBDV was high in backyard chickens in four flocks (105/119 or 88%; 81, 93%) and broilers on three farms (64/88 or 73%; 63, 81%) located in the same agricultural zone on Santa Cruz in 2005 [2]. The same study produced evidence that exposure to IBV was higher in study backyard chickens (95/119 or 80%; 72, 86%) compared to study broilers (9/88 or 10%; 5, 18%). In addition, the same study [2], investigated exposure to IBDV and other pathogens in 236 wild birds. All birds were classified as seronegative to pathogens tested including IBDV, NDV, and MG. In that study, one limitation was that serological testing in wild birds required pooling of samples, an inherent bias that could have produced false negative results if too few samples had positive antibody titers to selected pathogens. In the two previous studies [1,2] backyard chickens and broilers were sampled for detection of IBDV and IBV antibodies on one occasion. Thus, it was not possible to measure seroconversion to these two pathogens and produce serologic evidence of recent infection with IBDV or IBV in either population during the study periods.

In this study, one farm on Santa Cruz had one broiler classified as seropositive to NDV. It is possible this is a false positive result because the antibody concentration against NDV in the study broiler was low (i.e., S/P ratio = 0.25), and no other broilers tested seropositive or showed clinical signs associated with NDV infection. Limited funding prevented further testing to reduce false positive results (e.g., testing in series using the HI test, or the use of PCR methods or virus isolation to confirm diagnosis). A previous study produced evidence that exposure to NDV was 16/72 or 22% (14, 33%) on three of five farms in November 2003, and 0/27 or 0% (0, 13%) in backyard chickens on San Cristobal during 2001–2003 [1]. In that study, a sample size of 27 backyard chickens was not sufficient to detect exposure to NDV if prevalence of exposure was less than 10% [5]. Another study produced evidence that exposure to NDV was high both in study backyard chickens (53/119 or 45%; 95% CI = 36, 54%) in four flocks and broilers (32/88 or 36%; 27, 44%) on three farms in Santa Cruz in 2005 [2]. In that study, exposure to a field strain of NDV explained positive antibody titers detected in backyard chickens, whereas vaccine titers or exposure to a field viral strain explained observed exposure to NDV in broilers.

In our study, all broilers were classified as seronegative to MG. One explanation can be that study broilers were not exposed to this pathogen via (i) vertical (transovarian) transmission or (ii) indirect transmission between infected backyard chickens and susceptible broilers (e.g., contaminated feed or water, shoes, or equipment used by farm personnel). Another explanation can be that the burden of exposure to MG was low (e.g., < 5%), and both the cross-sectional sampling approach (broilers were sampled one time only) and the sample size of 10 broilers per farm were not sufficient to detect low levels of exposure to MG [5]. Previous studies have reported evidence of exposure to MG in broiler chickens on the Galapagos. In the study by Gottdenker et al. [1] 5/12 or 42% (19, 68%) broilers on one farm were classified as seropositive, and 60 broilers on four farms were classified as seronegative to MG in San Cristobal in November 2003. In that same study, 2/19 or 11% (3, 31%) and 12/19 or 63% (41, 81%) backyard chickens on San Cristobal and Santa Cruz, respectively, had positive antibody titers to MG during 2001–2003. In the study by Soos et al. [2] on Santa Cruz, exposure to MG was higher in backyard chickens in four flocks (55/119 or 46%; 38, 55%), compared to broilers on three farms (3/88 or 3%; 1, 10%).

Two of six poultry farms on Santa Cruz and six of seven poultry farms on San Cristobal had evidence of exposure to *Eimeria* spp. Coccidia infections in poultry are not a health hazard to Galapagos wild birds due to the parasite high host specificity [6]. Although, the source of *Eimeria* spp. infection in study farms is not known, one potential source is indirect transmission between infected backyard chickens (e.g., via farm personnel or use of equipment contaminated with *Eimeria* spp.) and susceptible broilers. In a previous study, *Eimeria* spp. infection was diagnosed in 16/68 (24%) backyard chickens on Santa Cruz and San Cristobal during July 2001–September 2003 [1]. In that study, 100 broilers were included, but they were not examined for intestinal parasites. In another study [2], 90 broilers, 120 backyard chickens, and 338 wild birds were investigated to measure exposure to multiple pathogens on Santa Cruz in June 2005. Fecal samples were collected from study chickens and wild birds, but samples were not tested for diagnosis of intestinal parasites. A third study [7] included 175 backyard chickens and 274 wild birds to measure exposure to multiple pathogens on Floreana Island during April-May 2008. In that study, fecal samples were collected from 63 wild birds, and six (10%) were diagnosed with *Isospora* sp. In that study, however, fecal samples were not collected from backyard chickens for diagnosis of intestinal parasites.

Two of 26 harvested broilers (Farm A and Farm F) had intestinal tract lesions. One broiler had mild lesions of the proximal duodenal mucosa consistent with *Eimeria acervulina* infection, and the second broiler had presence of blood and mucus in the cecum consistent with *Eimeria tenella* infection. In poultry, *Eimeria acervulina*, *Eimeria tenella*, and *Eimeria maxima* are the most commonly recognized species in broilers [8]. The effects of *Eimeria* spp. infection on poultry include decreased body weight gain, decreased feed efficiency, and increased maintenance energy costs [9,10]. In addition, infection with *Eimeria* spp. is a predisposing factor for necrotic enteritis and other intestinal infections in poultry due to its ability to cause mucosal damage [11]. Pathogenic *Eimeria* spp. have the potential to move quickly through a poultry pen and kill a large number of birds [12]. To our knowledge, this is the first study that has produced evidence of exposure to *Eimeria* spp. in broilers on the Galapagos.

This study had several limitations. First, the selection of six of 25 broiler farms on Santa Cruz was not random. Thus, the study results on Santa Cruz apply to study farms only. Second, the sample size of 10 broilers per farm was not sufficient to detect one or more broilers as exposed to MG if the burden of exposure per farm was less than 25%. Third, study broilers were blood sampled for detection of selected pathogens on one occasion. Thus, it was not possible to measure seroconversion to these pathogens and produce serologic evidence of recent infection in study broilers. Fourth, detection of antibodies to investigated pathogens was based on one serologic test (IDEXX ELISA) only. Testing in series or testing in parallel (using a second serologic test) could have reduce the risk of potential false positive or false negative results, respectively. Fifth, diagnosis of *Eimeria* was limited to oocyst morphology. Collection of intestinal scrapings would have been useful for a more accurate diagnosis coccidial infection in study broilers. Finally, backyard chickens were not included in this study, so it was not possible to measure and compare the burden of exposure to investigated pathogens between broilers and backyard chickens.

### Policy options

In order to mitigate the risk of disease transmission between broilers, backyard poultry, and wild birds, Galapagos’ policymakers can consider implementing a certification program (e.g., Green List, Red List) that requires poultry products to come from broiler chicken farms that follow Best Management Practices, including enhanced surveillance and biosecurity measures. For example: (i) use of health records to monitor morbidity and mortality events in broilers; (ii) use of written biosecurity protocols; (iii) use of peripheral fence to keep backyard chickens or other animals out; (iv) poultry pens protected against rodents or native birds; and (v) proper disposal of broiler pen bedding material.

All-in all-out management practices allow simultaneous depopulation of facilities between flocks, and time for periodic cleaning and disinfection to break the cycle of diseases. The implementation of this practice in small poultry operations on the Galapagos, however, can be difficult. Poultry farmers are expected to deliver chilled or frozen chicken meat in the local market (e.g., meat shops, grocery stores, restaurants, tourist boats) once or twice every week. The local business environment justifies the implementation of enhanced surveillance and biosecurity measures identified above.

## Supporting information

S1 GPS Data (XLS)
